# Bringing evolution into zoological parks through timetrees

**DOI:** 10.3389/fbinf.2026.1932369

**Published:** 2026-09-21

**Authors:** Jack M. Craig, Michael Suleski, S. Blair Hedges, Sudhir Kumar

**Affiliations:** 1 Institute for Genomics and Evolutionary Medicine, Temple University, Philadelphia, PA, United States; 2 Department of Biology, Temple University, Philadelphia, PA, United States; 3 Center for Biodiversity, Temple University, Philadelphia, PA, United States

**Keywords:** dating, outreach, phylogeny, subspecies, zoological parks

## Abstract

Zoological parks provide one of the public’s earliest and most frequent encounters with biodiversity, making them a natural setting for introducing evolution to the general public. Timetrees are particularly well suited to this purpose because they link the diversity of living animals to the history of life on Earth, but zoos rarely make this connection. Here, we present a new phylogeny and an associated open web resource that fill this gap (https://www.timetree.org/zootree). Using an intuitive interface, users can create educational outreach materials custom-tailored to the zoo collection of their choice. The timetree of zoo mammals will, therefore, offer the general public an opportunity to understand evolution through the lens of charismatic animal ambassadors.

## Introduction

Most members of the general public will only ever encounter charismatic animals like gorillas and elephants at zoological parks, creating a singular opportunity for education and outreach (Gusset and Dick, 2011; Miranda et al., 2023). This also makes zoos a natural setting for introducing evolution to the general public. Yet we find that zoos rarely take the opportunity to do so explicitly. Timetrees would be suitable tools for linking biodiversity patterns to the process of evolution, but no readily accessible resource is available to generate timetrees tailored to zoo collections. In particular, zoos frequently exhibit geographically distinct populations, often recognized as subspecies, that can be of conservation importance. Therefore, a timetree resolved at the subspecies level is needed to communicate the distinctiveness of the local forms to the visitors. For example, visitors encounter western gorillas (*Gorilla gorilla*) rather than simply gorillas, Indian elephants (*Elephas maximus indicus*) rather than Asian elephants, and many other regional forms that differ in morphology, ecology, and evolutionary history.

Most existing online timetree resources focus on species, offering no information on local varieties. Presenting only species-level relationships therefore omits the local diversity highlighted by zoological parks. This creates a marked gap as zoos cannot showcase their animal collections in the context of local conservation and evolutionary history. A subspecies-level timetree will enable visitors to understand how these local lineages are related to one another, how recently they diverged, and how they fit within the broader evolutionary history of mammals. By linking regional variation to deeper species divergences, such timetrees would illustrate that evolution continuously generates biodiversity across multiple hierarchical levels, from subspecies to species and higher taxa.

Accordingly, we have assembled a timetree of 423 mammals from zoos worldwide, because mammals are among the most familiar and effective taxa for public outreach and education. This timetree captures not only relationships among taxa but also the timing of their diversification, extending the evolutionary perspective available to zoo visitors. Uniquely, it spans deep mammalian divergences from the early Cretaceous to the Pleistocene while also including 201 subspecies across 50 species, all derived from published molecular phylogenies, an unprecedented combination of evolutionary breadth and depth that enables further scientific investigation. We make this timetree freely available at https://www.timetree.org/zootree, where users can explore the mammalian tree together with relevant Earth history information. This resource will help educators, visitors, and curators place zoo animals within the broader narrative of evolutionary history and, thereby, strengthen public engagement with the origins and conservation of biodiversity across geological time.

## Results

### A global phylogeny of mammals found in zoos

To build our timetree of zoo mammals, we assembled a comprehensive target species list from three sources with complementary scopes and purposes. First, the review publication Brereton and Brereton (2020), which summarized data from the International Zoo Yearbook’s list of ‘Zoos and Aquariums of the World’ from 1960 to 2018 (itself unavailable in digital form). Second, the database Zootierliste (www.zootierliste.de) maintains a record of all vertebrate species currently or previously housed in zoos associated with the European Association of Zoos and Aquaria (EAZA). Finally, A-Z Animals (https://a-z-animals.com/) is an educational resource with records of 852 mammal taxa that visitors can see at zoos. Together, these three sources contained 317 unique mammal species after filtering out hybrids, redundant common names (e.g., farm animals and dog breeds), and other non-mammal species.

Our survey of these resources revealed that many zoo mammals represent regionally distributed subspecific lineages, as denoted by trinomial scientific names (e.g., *Gorilla gorilla*), so we aimed to include all subspecies described for our 317 zoo mammal species. We queried the NCBI taxonomy and identified 84 species with named subspecies. While taxonomists do not uniformly accept subspecies as distinct evolutionary units, and the ICZN offers no guidance on their intended use, they are widely used for mammals where they often denote regional variants such as the western gorilla and Indian elephant. Since the application of the subspecies rank is uneven and often debated, we took the most conservative approach by retaining only the binomial species when one or more published phylogenies either directly refuted or synonymized the associated subspecies, or when they were omitted due to stated taxonomic uncertainty. Adding the remaining subspecies resulted in a curated target list of 553 taxa (Supplementary Material S1). This list served as the basis for our effort to reconstruct the phylogeny of zoo mammals.

To build a supertimetree containing all the subspecies, we used a multifaceted literature search and phylogenetic synthesis approach following Craig et al. (2023), Craig et al. (2024). First, we accessed the TimeTree of Life database version 5 (TT5) (Kumar et al., 2024) and obtained a backbone mammalian phylogeny that included 252 of our 553 target taxa. None of these were subspecies, as the TimeTree resource is primarily at the species level, but using subspecific substitutions (see *Methods*) we were able to add 44 more taxa for which TT5 had specific records, for a total of 296 backbone taxa. To account for the taxa still missing from the target list, we identified all published timed phylogenies that included at least one of the missing mammalian taxa (species or subspecies), resulting in 33 timed trees. Where computable Newick tree files were not provided, we transcribed the published phylogenies (topologies and branch lengths) from the figures included in the source publications. We used ImageJ (Schneider et al., 2012) to trace each tree exactly as it appears in print, then scaled each tree using the provided scale bar. This process has the benefit of precisely reproducing a phylogenetic hypothesis that would otherwise be inaccessible to modern bioinformatic methods, precluding redundant phylogenetic efforts and broadening the field’s evolutionary consensus. These timetrees contributed a total of 81 taxa, leaving 176 taxa still missing.

To incorporate species not present in the TT5 database or in a published timed phylogeny, we next searched for untimed phylogenies that included the remaining taxa, yielding 40 additional trees with 100 taxa. As with timed trees, these were perfectly reproduced as Newick files where needed, then we estimated divergence times by imposing literature consensus secondary calibrations as in previous work (Craig et al., 2023; Craig et al., 2024) and timed them using RelTime (Tamura et al., 2012; Tamura et al., 2018) in MEGA 12 (Kumar et al., 2024). Between the 33 timetrees, 40 newly timed trees, and subspecific substitutions, we added a total of nine species and 201 subspecies. This resulted in a total of 423 mammal taxa, including 288 unique species, of which 50 have multiple subspecies.

To synthesize these 73 phylogenies into a single supertimetree, we used Chrono-STA, which uses a shared chronological scale to overcome uneven taxonomic sampling and differences in evolutionary rate across a population of trees (Barba-Montoya et al., 2025) (Figure 1). With the addition of rich subspecific sampling, which was absent from the phylogenetic backbone, we also added a subspecies-level resolution supported by primary molecular taxonomic literature.

**FIGURE 1:**
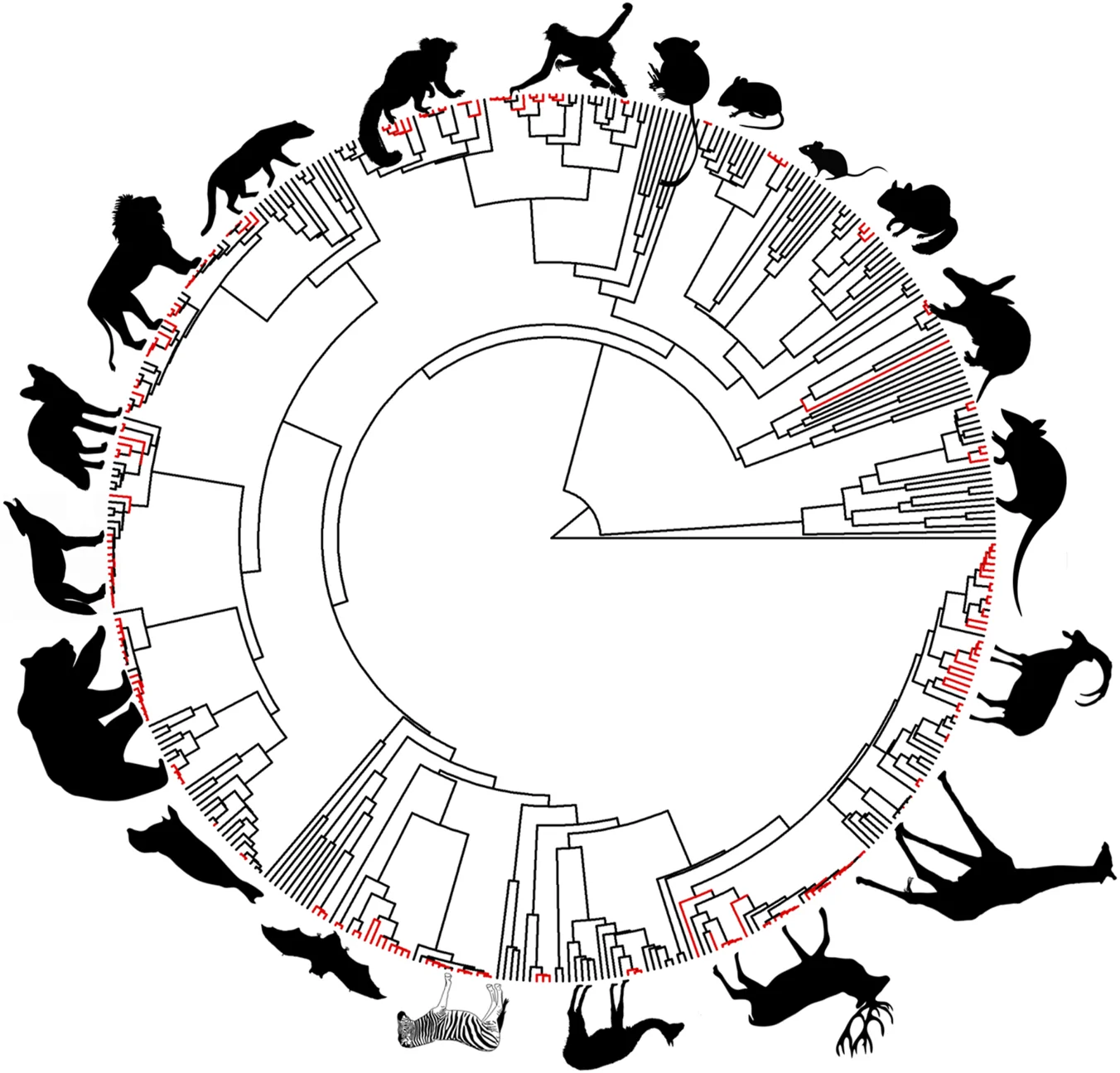
A phylogeny of mammals found in zoos. 423 mammalian taxa are represented, including 288 unique species. Taxa shown with red pendant edges were added to the TT5 backbone on the basis of published molecular phylogenies.

### Outreach and education

The phylogeny we have constructed here forms the basis of a new resource available at https://www.timetree.org/zootree. We will present the entire phylogeny of mammals in zoos, including all subspecies, with simple checkboxes for each tip and internal phylogenetic node (Figure 2A). Users will be able to check or uncheck each one (phylogenetic tips descendant from an unchecked node will automatically also be unchecked) to quickly and intuitively subset the total tree to just their desired composition, typically that of the zoo they either maintain or wish to visit (Figure 2B). Crucially, even simply observing and selecting their taxa of interest in a phylogenetic format rather than a simple alphabetical list may impart a new evolutionary perspective. Given their desired subset of taxa, users can export several helpful resources tailored to their selected mammals.

**FIGURE 2:**
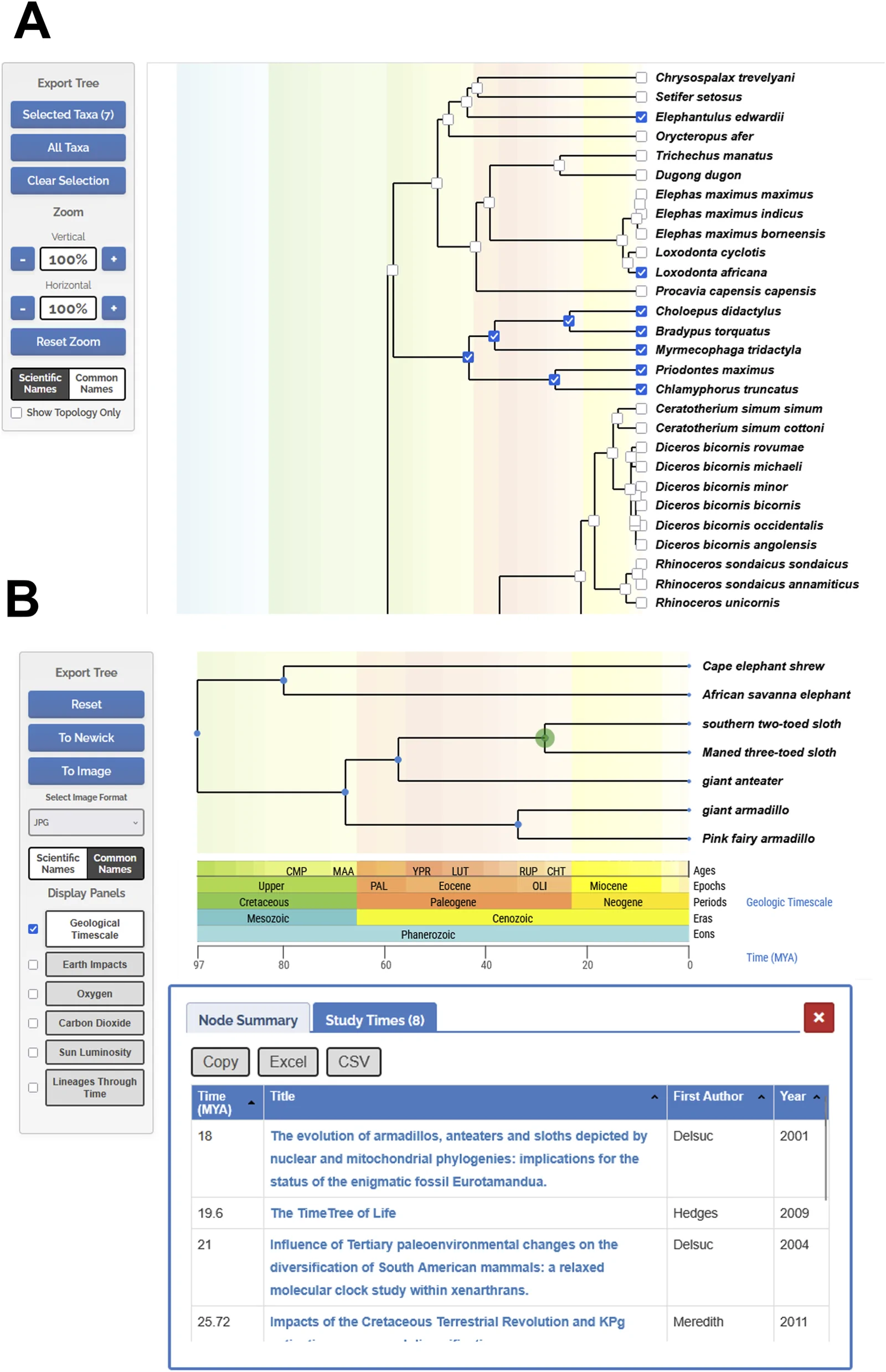
User interface of the Zoo TimeTree webpage. **(A)** Users may select the taxa present at their zoo of interest using a simple checkbox interface integrated into the familiar TimeTree viewer. **(B)** Users then focus on the selected tree where they can view all published time estimates for each node to determine the community consensus age, toggle freely between scientific and common names, and export their selected tree as a vector image or a Newick file for use in graphical outreach or research applications. All divergence times that contribute to the consensus time for a given node can be viewed in the associated timetable, illustrating any timing uncertainty.

## Conclusion

The timetree of mammals found in zoos contains 423 mammal taxa, including 288 unique species. This alone represents a charismatic subset of biodiversity, making it ideal for outreach and evolutionary education. It also contains 201 subspecific taxa, representing the largest phylogenetic breadth of subspecific diversity given that many phylogenies incorporating subspecies are narrowly focused at the genus level or below. Thus, this work establishes not only a valuable outreach tool for zoo visitors to connect biodiversity patterns with macroevolutionary processes, but also an opportunity for researchers to investigate subspecific evolution across taxa.

For example, we found that while two species had over ten subspecies each in our tree (the wolf, *Canis lupus*, and the red deer, *Cervus elephas*), there were only a further five with more than five subspecies each, 41 species with only two subspecies, and 238 with none at all (Figure 3A). Indeed, the pattern of biodiversity below the species level fits a distribution common to other higher taxonomic ranks (McGill et al., 2007) (Figures 3B–D). Likewise, for all species with two or more subspecies, we observed an average subspecific crown age of 2.8 million years (My) (Figure 3E). This is consistent with a similar inference from several major clades of the tree of life, which inferred times to speciation of 2.1 My in vertebrates, 2.2 My in arthropods, and 2.7 My in plants (Hedges et al., 2015). Thus, the evolution of subspecies shares patterns with that of species, lending credence to their use as biological clades, an insight made possible only by constructing the timetree of species.

**FIGURE 3:**
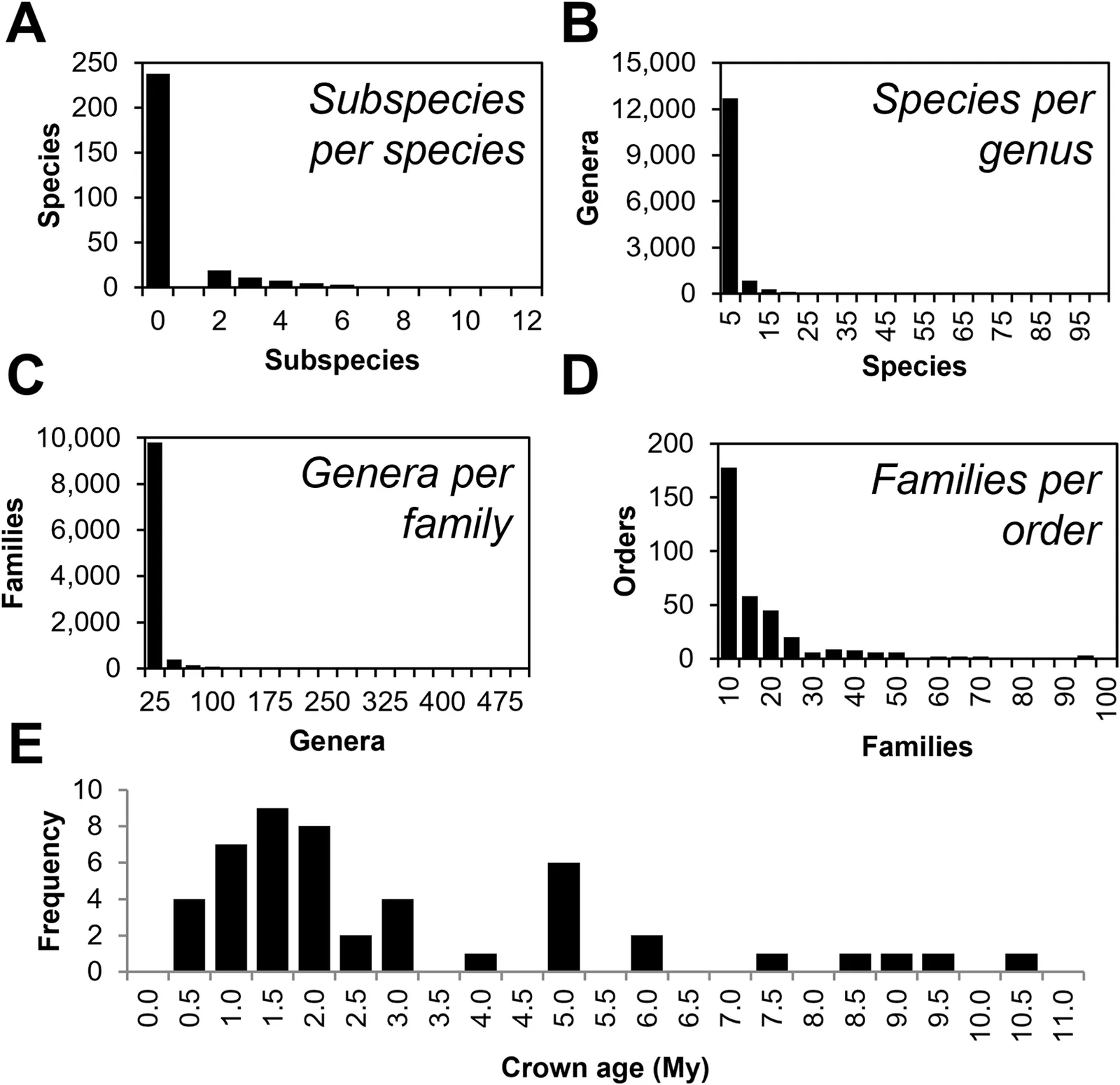
The distribution of taxa per taxonomic rank. **(A)** The majority of species have 0–2 subspecies. **(B)** The majority of genera have five or fewer species. **(C)** The majority of families have 25 or fewer genera. **(D)** The majority of orders have ten or fewer families (1,593 orders with five or fewer species not shown). **(E)** Average crown age of each species with two or more described subspecies was 2.8 My.

## Methodological details

### Taxonomic reference

To define the list of species which occur in zoos worldwide, we drew on three sources: Brereton and Brereton (2020), which summarized data from the International Zoo Yearbook’s list of “Zoos and Aquariums of the World” from 1960 to 2018, Zootierliste (www.zootierliste.de), which maintains a record of all vertebrate species maintained currently or previously in zoos associated with the European Association of Zoos and Aquaria (EAZA), and A-Z Animals (https://a-z-animals.com/), an educational resource with records of 852 mammal taxa, especially subspecies. We combined all unique names from these three sources, pruned non-mammal taxa, and then matched each species name to the NCBI taxonomy database (Schoch et al., 2020).

Unmatched taxa, including hybrids and domestic breeds such as dogs and farm animals, were also dropped, leaving only binomial species names that occurred in any of our three primary sources and had a matching NCBI taxon ID. We then augmented this list of species by adding any associated subspecies present in the NCBI taxonomy database (binomial species names were excluded when one or more trinomial subspecies names were available). NCBI had 2,061 trinomial subspecies names in mammals, which we pared down to only those corresponding to our target species list. Since substantial disagreement exists among taxonomists regarding the validity of subspecies, we then further discarded any trinomials when we found conflicting taxonomic records in contemporary publications, omission from accounts of diversity within the corresponding species, or explicit rejection of the taxon. In these cases, we retain only the binomial to ensure the most conservative possible taxon list.

This resulted in a final taxon list of 553 mammals (Supplementary Material S1), which served as the focus of our data search and tree-building efforts. Since the subspecies rank is not universally accepted and is afforded only minimal consideration by the ICZN, there can be considerable taxonomic discord surrounding the validity of some subspecies. A rigorous taxonomic reevaluation complete with formal elevations or synonymizations is outside the scope of this phylogenetic project. Hence, in cases where the validity of certain subspecies was contested, we adopt a conservative approach by including only the binomial.

### Phylogenetic backbone

We used the fifth edition of the TimeTree of Life (TT5) database’s super-timetree as the phylogenetic backbone in our study (Kumar et al., 2022). First, we extracted the timetree of all mammals from their global supertimetree. Then, for each species binomial in the backbone for which we had one or more subspecies in our taxonomic reference, we renamed the associated tip in the backbone to reflect the type subspecies name. This created anchoring points where subspecies trees could be merged with the backbone based on taxonomic overlap, which would not be possible otherwise, as TimeTree does not include subspecific diversity at this time. Importantly, as all subspecies of a given species are equally distantly related to any other species on the tree, this name change does not affect our topology or estimated divergence times; it only enables supertree building.

### Phylogeny search and processing

Our literature search produced 33 published, time-calibrated phylogenies (Cortés-Ortiz et al., 2003; Pitra et al., 2004; Kurose et al., 2005; Barnett et al., 2009; Lavergne et al., 2010; Gonder et al., 2011; Frankham et al., 2012; Zhang and Zhang, 2012; Vilstrup et al., 2013; Hopken et al., 2015; Morales-Jimenez et al., 2015; Puckett et al., 2015; Li et al., 2016; Radu et al., 2016; Grobler et al., 2017; Maswanganye et al., 2017; Eldridge et al., 2018; Tumendemberel et al., 2019; Dussex et al., 2020; Joshi et al., 2020; Rai et al., 2020; Stanton et al., 2020; van der Valk et al., 2020; Allio et al., 2021; Spitsyn et al., 2021; Cai et al., 2022; Elalouf et al., 2022; Mackiewicz et al., 2022; Pérez et al., 2022; Craig et al., 2023; Özkan et al., 2023; Sun et al., 2023), including five mammal species and 76 subspecies not included in our backbone tree. For cases where tree files in Newick or comparable formats were not readily available, we had to reproduce the printed figure. To do so, we first reconstructed the phylogeny manually using the topology editor in MEGA 12 (Kumar et al., 2024). We added branch lengths to these trees by capturing the precise lengths of each branch in pixels from the published figure using ImageJ (Schneider et al., 2012), then converting them to millions of years using the provided scale bar. These values were then added to the topology we drew in MEGA, and the resulting timetree was exported as a Newick string.

We also identified 40 additional phylogenies (Slattery and O’Brien, 1995; Randi et al., 2001; Sato et al., 2003; Fyler et al., 2005; Good et al., 2008; Kurose et al., 2008; Hafner et al., 2011; Ouma et al., 2011; Rueness et al., 2011; Hassanin et al., 2012; Macholán et al., 2012; Olivieri et al., 2014; Wilder et al., 2014; Vonhof et al., 2015; Wood and Grabowski, 2015; Ambriz-Morales et al., 2016; Ding et al., 2016; Fan et al., 2016; McLean et al., 2016; Githui et al., 2017; Glaubrecht and Neiber, 2017; Hua et al., 2017; Patel et al., 2017; Ruiz-García et al., 2017; Mizuno et al., 2018; Cho et al., 2019; Zhou et al., 2019; Ben Chehida et al., 2020; Hong et al., 2020; Kim et al., 2020; Moscardi et al., 2020; Naseem et al., 2020; Bit et al., 2021; Niedziałkowska et al., 2021; Costa et al., 2022; Herrera et al., 2022; Sooriyabandara et al., 2023; Sosale et al., 2023; Wang et al., 2023) that had not been timed, in which branch lengths were proportional to the number of molecular substitutions. These contributed a further 100 taxa. We time-calibrated these trees in RelTime (Tamura et al., 2012; Tamura et al., 2018) using an established secondary calibration approach (Craig et al., 2023; Craig et al., 2024) that relies on consensus divergence times from the literature (in this case, the TT5). This is especially useful for dating divergences in subspecific trees, as primary fossil calibrations are often unavailable or uninformative for very shallow divergences. We used a single calibration point for each phylogeny. Since RelTime estimates relative divergence times with respect to an outgroup, not all the trees we found had outgroups. We identified the basal split in each case, then timed the tree twice, taking one daughter of this split as the outgroup to its sister in each case. This resulted in two timed sister trees, each comprising half of the original tree’s diversity. All untimed trees, calibration schemes, and final timetrees for each case are available upon request.

### Super-timetree inference

We took the resulting population of 73 timed phylogenies and synthesized them using Chrono-STA. To ensure that Chrono-STA could fully merge our trees, we added two universal outgroup taxa at the root of each: the chicken (*Gallus)* and the zebrafish (*Danio rerio*). Based on TimeTree 5, these diverged from the lineage comprising all mammals 319 million years ago (median of 30 published estimates) and 429 million years ago (33 estimates), respectively (Kumar et al., 2022), making it simple to append these outgroups to our existing timed phylogenies. We ran Chrono-STA on the default parameters (Barba-Montoya et al., 2025), then pruned all extraneous taxa from the resulting supertree, including the chicken, zebrafish, and all other taxa not included in our taxonomic reference. This process ensures that the sample of timed molecular phylogenies is synthesized into a cohesive, literature-consensus, timed molecular phylogeny without the need for manual reconstruction or phylogenetic imputation.

## Data Availability

Publicly available datasets were analyzed in this study. This data can be found here: https://www.timetree.org/zootree.
